# ATG8-Interacting Motif: Evolution and Function in Selective Autophagy of Targeting Biological Processes

**DOI:** 10.3389/fpls.2021.783881

**Published:** 2021-11-29

**Authors:** Wanqing Liu, Zinan Liu, Zulong Mo, Shaoying Guo, Yunfeng Liu, Qingjun Xie

**Affiliations:** ^1^State Key Laboratory for Conservation and Utilization of Subtropical Agro-Bioresources, Guangdong Provincial Key Laboratory of Plant Molecular Breeding, South China Agricultural University, Guangzhou, China; ^2^Rice Research Institute, Guangdong Academy of Agricultural Sciences/Guangdong Key Laboratory of New Technology in Rice Breeding/Guangdong Rice Engineering Laboratory, Guangzhou, China; ^3^State Key Laboratory for Conservation and Utilization of Subtropical Agro-Bioresources, College of Life Science and Technology, Guangxi University, Nanning, China

**Keywords:** autophagy, ATG8, ATG8-interacting motif (AIM), ubiquitin-interacting motif (UIM), selective autophagy receptor (SAR)

## Abstract

Autophagy is an evolutionarily conserved vacuolar process functioning in the degradation of cellular components for reuse. In plants, autophagy is generally activated upon stress and its regulation is executed by numbers of *AuTophaGy-related genes* (*ATGs*), of which the ATG8 plays a dual role in both biogenesis of autophagosomes and recruitment of ATG8-interacting motif (AIM) anchored selective autophagy receptors (SARs). Such motif is either termed as AIM or ubiquitin-interacting motif (UIM), corresponding to the LC3-interacting region (LIR)/AIM docking site (LDS) or the UIM docking site (UDS) of ATG8, respectively. To date, dozens of AIM or UIM containing SARs have been characterized. However, the knowledge of these motifs is still obscured. In this review, we intend to summarize the current understanding of SAR proteins and discuss the conservation and diversification of the AIMs/UIMs, expectantly providing new insights into the evolution of them in various biological processes in plants.

## Introduction

Being unable to move, plants are often confronted with adversely stressful conditions, including abiotic and biotic stress (Zhu, 2016). Accordingly, a set of complicated cellular and metabolic responses for survival under such severe conditions have been evolved in plant cell, of which a highly conserved mechanism (termed autophagy, meaning “self-eating”) has been developed. Autophagy facilitates the vacuole-dependent (in plants and yeast) or lysosomes-dependent (in animals) degradation of unwanted cell components, and consequently activates the recycling of cellular material to provide stress relief (Bassham et al., 2006). To date, three types of autophagy have been well described in plants, including macroautophagy, microautophagy, and megaautophagy. Macroautophagy is involved in the delivery of cytoplasmic constituents by double-membrane vesicles (termed autophagosomes) to the lytic vacuoles for turnover. With respect to the microautophagy, cytoplasmic components are engulfed by invagination of the tonoplast. As an extreme form of autophagy, megaautophagy functions in the last stage of developmental programmed cell death (PCD), leading to the degradation of cytoplasmic components following the permeabilization or rupture of the tonoplast and release of vacuolar hydrolases (Marshall and Vierstra, 2018). Among them, the macroautophagy (hereafter referred to autophagy) is the most well-characterized one in plants.

The autophagic process was first demonstrated by Christian de Duve and his associates with electron microscopy (EM) studies (Klionsky, 2008). Due to the limitation of research approaches, the physiological functions and molecular mechanisms of autophagy have not been fully understood until the discovery of *AuTophaGy-related genes* (*ATGs*) in yeast (*Saccharomyces cerevisiae*) by Yoshinori Ohsumi in 1990s, earning him the 2016 Nobel Prize in Physiology and Medicine. To date, it has been revealed that *ATGs* function in each step of the autophagy machinery and their homologs are highly conserved in eukaryotes (Ohya et al., 1986; Mizushima et al., 2011; Yu et al., 2018). Attributing to the identification of *ATGs* homologs, more than 40 *ATG* genes were isolated among different plant species, including the *Arabidopsis* (*Arabidopsis thaliana*), tomato (*Solanum lycopersicum*), maize (*Zea mays*), and rice (*Oryza sativa*). Characterization of plant *ATGs* enabled further understanding of autophagy function in various biological processes (Doelling et al., 2002; Chung et al., 2009; Zhou et al., 2014; Wada et al., 2015). However, the identification of selective autophagy receptors (SARs) still remains further elusive.

## Autophagy Machinery in Plants

Autophagy can either selectively degrade specific cellular components or non-selectively degrade cytoplasm in bulk. In both cases, the cytoplasmic materials are devoured by a double membrane structure, namely autophagosome, which is afterward imported into vacuole for degradation. Briefly, autophagy is activated according to the nutritional and developmental status of the cell, and this induction is affected by the activity of several protein kinases, such as Target of Rapamycin (TOR) kinase. Activated TOR hyperphosphorylates the ATG1 partner, ATG13, to prevent the ATG1/ATG13 complex assembly, thereby, inhibiting autophagy. Upon TOR inactivation, dephosphorylation of ATG13 permits ATG1, ATG11, and ATG101 to form the active complex (Liu and Bassham, 2010; Suttangkakul et al., 2011; Li et al., 2014; Dobrenel et al., 2016; Pu et al., 2017). Meanwhile, the activated ATG1 kinase stimulates the ATG9/ATG2/ATG18 complex to recruit lipids to the expanding phagophore. The phosphatidylinositol-3-kinase (PI3K) complex composed of vacuolar protein sorting 34 (VPS34), VPS15, ATG6, and ATG14 or VPS38 subunit, generates phosphatidylinositol-3-phosphate (PI3P) to decorate the expanding phagophore (Marshall and Vierstra, 2018). This decoration process is accompanied by the attachment of ATG8 to phosphatidylethanolamine (PE) regulated by ATG5/ATG12/ATG16 E3 ligase complex (Romanov et al., 2012). Once inserted into the emerging phagophore, the ATG8-PE adduct helps in sealing the vesicle to generate a mature autophagosome (Marshall and Vierstra, 2018). Subsequently, the entire autophagosome is transported to the vacuole and its outer membrane is fused with the tonoplast with the help of Fab1, YOTB/ZK632.12, Vac1, and EEA1 (FYVE) domain protein required for endosomal sorting 1 (FREE1) and other components (Gao et al., 2014; Kolb et al., 2015; Kalinowska et al., 2018; Marshall and Vierstra, 2018). Finally, the autophagic body is deposited and its membrane and contents are degraded by vacuolar hydrolases, resulting in the breakdown and recycling of nutrients.

## The Classification of ATG8-Interacting Proteins

**FIGURE 1:**
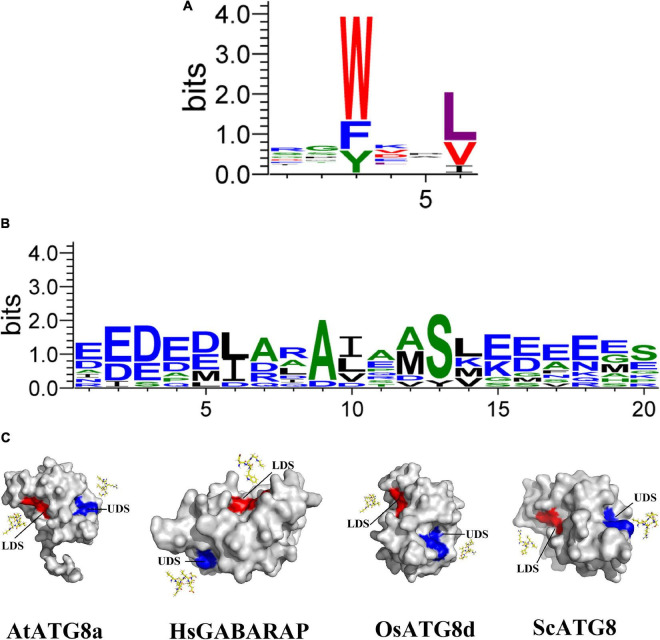
Structures and binding motifs of the UIM docking site and the LIR/AIM docking site proteins. **(A)** The plant AIM consensus sequences are identified by WebLogo by using the known AIMs as shown in Table 2. **(B)** The plant UIM consensus sequences are identified by WebLogo by using the known AIMs as shown in Table 1. **(C)** Surface view of the three-dimensional structure of four ATG8 proteins corresponding to *Arabidopsis thaliana*, *Homo sapiens*, *Oryza sativa*, and *Saccharomyces*. Eight AIM-containing peptides shown in yellow stick form. Blue and red shading represent UDS and LDS, respectively. The structure of four proteins is derived from SWISS-MODEL database (Guex et al., 2009; Bertoni et al., 2017; Bienert et al., 2017; Waterhouse et al., 2018; Studer et al., 2020).

In addition to the contribution to autophagosome formation mentioned above, ATG8 plays an additional key role in the selection of specific SARs to be sequestered prior to its degradation. In this process, ATG8 proteins bind to these SARs *via* their ATG8-interacting motifs [AIMs or LC3-interacting regions (LIRs) in animals] or ubiquitin-interacting motifs (UIMs; Farré and Subramani, 2016; Dikic, 2017; Marshall and Vierstra, 2018; Figures 1A,B), indicating that AIM/UIM is the core apparatus linking selective autophagy to its targets. Even though there is less conservation among these SAR proteins, a core consensus sequence consisting of W/F/Y-X-X-L/V/I regarding to AIM is identified (Figure 1A), which often prefers the surrounding of one or more acidic residues within W or L site (Xie et al., 2016). To bind to the W- and L-sites, the LC3-interacting region (LIR)/AIM docking site (LDS) of ATG8 is usually present in an extended β-conformation with exposed hydrophobic side chains (Figure 1C). If potential AIM residues are buried within the molecule, it might be incapable of interacting with ATG8 (Noda et al., 2008, 2010). As a consequence, simple identification of AIM based on this consensus sequence may bring false positive candidate, which still needs to be experimentally validated (Kalvari et al., 2014; Xie et al., 2016). To address this issue, at least two bioinformatic approaches have been developed by introducing stringent criteria (Kalvari et al., 2014; Xie et al., 2016), definitely convincing a high-throughput analysis of AIM in various organisms. With respect to the UIM, it presents in another surface of ATG8 (Marshall et al., 2019; Figure 1C). Notably, the UIM docking site (UDS) is consisted of less residues than that of LDS, Ψ-F-Ψ-Ω/T (Marshall et al., 2019), implying a less spectrum of UIM proteins. In fact, only a few of UIM-containing SARs has been identified so that it is difficult to generate a reliable regular pattern of its consensus sequences (Figure 1B and Table 1), thereby hampering the genome-wide *in silico* high throughout the identification of UIM.

**TABLE 1:** Known ATG8-interacting proteins in plant.

Species^a^	Protein name (Locus^b^)	Pattern^c^	PSSM Score^d^	Annotation	References
*A. thaliana*	DSK2(NP_565407.1)	ESFKEL	10	Degradation of brassinosteroid-responsive transcription factor BES1	Zhao et al., 2002; Jamet et al., 2008; Kaur et al., 2013; Nolan et al., 2017
		EGFNML	10		
		RMYENV	10		
	ABS3(NP_194643.1)	RGWAPL	15	Encodes Activated Disease Susceptibility 1	Wang et al., 2015; Jia et al., 2019
		GLWVGL	12		
	RGS1(NP_189238.2)	SDYVAV	16	Unknown	Ghusinga et al., 2021; Suo et al., 2021
		KSYIFL	12		
		SFWIPV	12		
		DLWKGI	12		
		ISWLQV	13		
		DYWSSI	14		
	GLK1(NP_565476.1)	IDFDDI	13	Unknown	Gommers et al., 2021; Zhao et al., 2021
		RPWLPL	15		
	KIN7.4(NP_195616.2)	DEYDGV	13	Unknown	Gommers et al., 2021; Zhao et al., 2021
		DKFDSL	12		
		NMWVLV	16		
	GLT1(NP_850828.1)	AGWFDL	14	Unknown	Baslam et al., 2017; Li et al., 2017
		FTWKAL	16		
	COILIN(NP_172762.2)	RSWVVL	20	Unknown	Kanno et al., 2020; Zheng et al., 2020
		IDYEQL	13		
		SPWEEL	17		
	NTR1(NP_173150.1)	ETWETL	20	Encodes a homolog of spliceosome disassembly factor NTR1. Required for correct expression and splicing of DOG1, a regulator of seed dormancy. The mRNA is cell-to-cell mobile.	Thieme et al., 2016; Wang et al., 2019
		QGWDPL	17		
		SSWRKL	15		
		HPWLPI	12		
		SPWKTV	13		
		TSWEQL	16		
		PKWLDV	14		
		YGWKEL	14		
		GGWFLV	13		
	PTR2(NP_178313.1)	IIWVPL	12	Unknown	Choi et al., 2020; Ye et al., 2021
	SUC3(NP_973404.1)	GPWDQL	14	Unknown	Chaparro et al., 2013; Xu et al., 2018
	CCI1(NP_201351.1)	GGFVVL	12	Unknown	Dal Bosco et al., 2004
*O. sativa*	DSK2a(XP_015614223.1)	RMYETV	13	Unknown	Wang et al., 2020
		EGFNML	10		
	ABS3(ABF94773.1)	RGYVPI	13	Unknown	Qin et al., 2021
		RGWPAL	14		
	RGS1(NA)	NA	NA	Unknown	In this study
	GLK1(BAD37323.1)	EGYDAV	12	Unknown	Ito and Kurata, 2006
		RLWSLL	12		
	KIN7.4(BAS96703.1)	NMWTVL	17	Unknown	In this study
	GLT1(BAS70238.1)	AGWLDL	17	Unknown	In this study
		FTWKAL	16		
	COILIN(BAT16766.1)	RCWLLL	14	Unknown	In this study
	NTR1(BAS90051.1)	DEFVTV	15	Unknown	In this study
	PTR2(BAS86116.1)	STFDVV	12	Unknown	Jie et al., 2010; Léran et al., 2014
		RGFTEL	12		
		IIWVPL	12	Unknown	
	SUC3(BAS82530.1)	SAWAAV	11	Unknown	Hirose et al., 2010; Siahpoosh et al., 2012
	CCI1(BAT05425.1)	GSFAVL	8	Unknown	In this study

*^a^A. thaliana, Arabidopsis thaliana; O. sativa, Oryza sativa.*

*^b^Gene Locus of the each protein is derived from TAIR (https://www.Arabidopsis.org/index.jsp) and China Rice Data Center (http://www.ricedata.cn/gene/).*

*^c^Pattern of the AIM is predicted by the iLIR online tool (https://ilir.warwick.ac.uk/index.php).*

*^d^Position Specific Scoring Matrix (PSSM) is a commonly used representation of motifs or patterns.*

The experimentally verified ATG8-interacting proteins can be mainly divided into four groups according to their predicted or known functions in the autophagic processes. The first group is referred to the autophagic proteins ATG1, ATG3, ATG4, and ATG7, which can directly regulate ATG8 during its lipidation with PE. ATG1 kinase complex, including the ATG13 and ATG101 regulatory subunits and the ATG11 scaffold protein, is involved in the regulation of the initiation of the preautophagosomal structure (PAS) formation. Both ATG1 and ATG11 directly bind ATG8 through the AIM REYVLV in ATG1a, and DNFDDI and CEYFIV in ATG11, respectively (Li et al., 2014). These interactions may not only stimulate the direct PAS assembly but also promote its autophagic turnover in which the ATG1a is delivered to the vacuole with ATG8-decorated autophagic bodies (Suttangkakul et al., 2011; Kraft et al., 2012; Li et al., 2014). Notably, the presence of ATG11 promotes starvation-induced phosphorylation of ATG1 and turnover of ATG1 and ATG13. Therefore, it is presumed that the breakdown of ATG1 kinase complex leads to the suppression of starvation-induced autophagy by removing this central regulator (Suttangkakul et al., 2011; Li et al., 2014). Cysteine proteinase ATG4 is mostly involved in the processing and delipidation of ubiquitin-like ATG8 proteins. Two homologs of the yeast *ATG4* gene have been identified in *Arabidopsis*, the *AtATG4a* and *AtATG4b*. Among them, AtATG4b has been experimentally validated to interact with two AtATG8 isoforms, AtATG8a and AtATG8d. Recent studies showed that AtATG4a and AtATG4b have different enzyme activity and exhibit different substrate affinity. AtATG4a predominantly cleaves AtATG8a, AtATG8c, AtATG8d, and AtATG8i, whereas other AtATG8 isoforms are processed by AtATG4a/b with similar efficiency. However, *atatg4a* is more sensitive to H_2_O_2_ than *atatg4b* under high H_2_O_2_ concentration, suggesting that plant might use *AtATG4b* to activate autophagy in response to oxidative stress (Ketelaar et al., 2004; Yoshimoto et al., 2004; Park et al., 2014; Woo et al., 2014).

The second group of ATG8-interacting proteins includes the Bin/Amphiphysin/Rvs (BAR) domain- and Src homology-3 (SH3) domain-containing family proteins that are involved in stimulating the development and closure of phagophore during autophagosome maturation (Behrends et al., 2010). SH3 domain-containing protein 2 (SH3P2) is a member of this family and localized on the autophagosomal membrane. *SH3P2*-RNA interference plant contained less autophagosomes and autophagic bodies during autophagy-induced conditions, further suggesting that SH3P2 positively regulates autophagosome formation. In addition, SH3P2 is proved to have membrane binding capability and specifically bind to PI3P. Moreover, further interaction analysis demonstrated that SH3P2 associates with the PI3K complex components, ATG6 and VPS34, as well as directly interacts with ATG8, thereby modulating autophagy activity (Zhuang et al., 2013; Zhuang and Jiang, 2014).

The third group of ATG8-interacting proteins is represented by the FYVE and coiled coil domain containing adaptor proteins 1 (FYCO1) (Pankiv and Johansen, 2010). FYCO1 proteins have been shown to interact with ATG8 and PI3P on autophagosomes, as well as with RAB7 Ras-bound GTPases to translocate autophagosomes (Pankiv et al., 2010). Nine FYCO1 homologs have been identified in *Arabidopsis* (Wywial and Singh, 2010), but it is still unknown whether other homologs are also involved in autophagosome transport in plants.

The last group of ATG8-interacting proteins is briefly referred to as the SARs, which participate in recruiting autophagy substrates. We will extent the discussion of the evolution of the AIMs/UIMs that are anchored SARs, as well as the SARs-mediated various types of selective autophagy in relevant biological processes between *Arabidopsis* and rice in the following.

## Involvement of ATG8-Interacting Motif Proteins in Various Types of Selective Autophagy

### ATG8-Interacting Motif Proteins in Xenophagy

**FIGURE 2:**
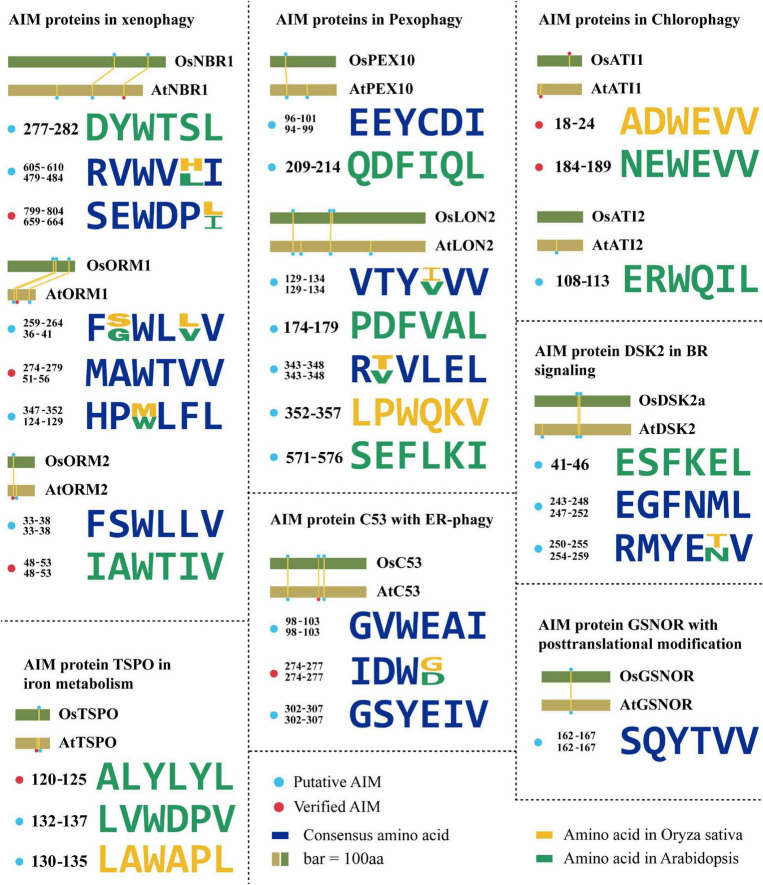
The evolution of AIMs derived from the known ATG8-interacting proteins in plants. Degenerate sequences of AIMs of known ATG8-interacting proteins by which AIMs in *Arabidopsis thaliana* and *Oryza sativa* are shown in yellow and green, respectively. Blue sequences are consensus amino acids of AIMs in both species. The rice AIM containing proteins are derived from alignments of *Arabidopsis* proteins known to interact with ATG8 from the NCBI database (https://blast.ncbi.nlm.nih.gov/). AtNBR1 (Q9SB64.1), OsNBR1 (KAF2945618.1), AtORM1 (NP_563622.1), OsORM1 (XP_015627600.1), AtORM2 (NP_199015.1), OsORM2 (XP_015637165.1), AtDSK2 (NP_565407.1), OsDSK2 (XP_015614223.1), AtPEX10 (NP_001323820.1), OsPEX10 (BAG87060.1), AtPEX6 (NP_171799.2), OsPEX6 (XP_015634963.1), AtLON2 (NP_568675.1), OsLON2 (BAD33324.1), AtC53 (NP_196301.2), OsC53 (XP_015644258.1), AtATI1 (NP_566059.1), OsATI1 (NP_001052131.1), AtATI2 (NP_567174.1), OsATI2 (NP_001048554.1), AtGSNOR (NP_001190468.1), OsGSNOR (XP_015627169.1), AtTSPO (NP_566110.1), OsTSPO (XP_015639220.1).

The Neighbor of BRCA 1 (NBR1) has been markedly documented as a master SAR of xenophagy, aiding in pathogen defense using either pro-death or pro-survival strategies (Hofius et al., 2017). Plant NBR1 is a chimeric protein performing integrative functions of two mammalian autophagy receptors, p62 and NBR1. NBR1 has been known to bind the viral capsid protein and particles of cauliflower mosaic virus (CaMV), leading to their autophagic degradation, thereby arresting the CaMV infection. This antiviral xenophagy could be offset by protective functions of autophagy-resistant CaMV inclusion bodies (Hafrén et al., 2017). Interestingly, a virus is also ingenious to evolve a strategy to utilize autophagy to protect itself. For example, the turnip mosaic virus (TuMV) could induce unfolded protein response (UPR)-dependent NBR1-ATG8 autophagy, and then release the viral RNA-dependent RNA polymerase (RdRp) of the virus replication complex (VRC), termed NIb, to form complex with NBR1-ATG8, eventually facilitating the VRC into the tonoplast for enhancing virus propagation (Li et al., 2020). AtNBR1 binds AtATG8 through a conserved AIM motif SEWDPI (Svenning et al., 2011; Figure 2). Notably, two homologous NBR1 are existed in rice (Zhang and Chen, 2020), which also possesses numerous predicted AIMs by iLIR analysis, showing that the AIM with SEWDPL residues is almost identical to that of AtNBR1 (Figure 2). Based on the sequence similarity of NBR1 AIMs between *Arabidopsis* and rice, we speculated that OsNBR1 may be involved in the xenophagy to defend the virus attack. Considering two NBR1 components in mammalian cell, the p62 and NBR1, it therefore raises an possibility for the evolution of two OsNBR1 homologs in rice by which they may function as cofactors in xenophagy or functionally divergent in response to different virus.

*Arabidopsis* orosomucoid (ORM) proteins (ORM1 and ORM2) acting as negative regulators of sphingolipid biosynthesis are thought to function in mediating xenophagy (Breslow et al., 2010; Han et al., 2010). It has been experimentally validated that both ORM1 and ORM2 can interact with ATG8 and that their respective N-terminal AIMs (WTVV and WTIV) are required for binding ATG8 (Han et al., 2010; Figure 2). ORM proteins modulate plant immunity by regulating pattern recognition receptor FLAGELLIN SENSING 2 (FLS2) protein accumulation. Reduced expression or null mutation of *ORM1/2* specifically enhanced FLS2-dependent immune responses after infection by bacterial pathogen *Pseudomonas syringae* and increased the abundance of FLS2, while overexpression of *ORMs* caused FLS2 degradation and abrogated FLS2-dependent signaling, suggesting a broader role of ORM proteins beyond sphingolipid metabolic regulation (Yang et al., 2019). Interestingly, two rice ORM1 homologs were identified in the homology searches using AtORM1 as a query (Kimberlin et al., 2016), and the conserved AIM (WXXV) is also present in these two OsORM1 proteins at their N terminus (Figure 2), suggesting that both of them may act as similar SAR in xenophagy. It is unclear why and how the two homologs evolved in rice, characterization of the difference of the express patterns between them may provide a cue for addressing this issue, particularly the specific role of them in xenophagy upon various viruses.

### ATG8-Interacting Motif Proteins in Aggrephagy

In addition to the role in xenophagy, NBR1 is also involved in the aggrephagy, which turns over the misfolded proteins caused by mutations, incomplete translations, misfolding after translation, aberrant protein modifications, oxidative damage, and ill-formed protein complexes (Sun et al., 2020). In *Arabidopsis*, AtNBR1 has two Ubiquitin-Associated (UBA) domains, but only the C-terminal UBA domain can bind ubiquitin. Both *Arabidopsis* and tomato *nbr1* mutants were hypersensitive to heat and oxidative stress, and ubiquitinated protein aggregates were highly accumulated in them (Zhou et al., 2013, 2014), suggesting that NBR1 is functionally conserved in aggrephagy among different plant species. However, the exact regulatory mechanism of NBR1-mediated aggrephagy in plants is still unclear. A recent report in human pathology revealed that the aggregation of ubiquitinated proteins by NBR1 was regulated by the Glycogen Synthase Kinase 3 (GSK3)-mediated phosphorylation of NBR1 (Nicot et al., 2014), suggesting NBR1-dependent aggrephagy is determined by posttranslational modification of its protein status. This regulation is also deserved to be verified in different plants, as well as the upstream signal of NBR1, such as corresponding kinase. Correspondingly, the OsNBR1 also seems to perform similarly as the highly conserved AIMs present (Figure 2).

### ATG8-Interacting Motif Proteins in Pexophagy

A selective degradation of peroxisomes by autophagy (termed as pexophagy) has been documented in plants (Farmer et al., 2013; Kim et al., 2013; Shibata et al., 2014). In this process, oxidized peroxisomes caused by H_2_O_2_ treatment were aggregated and broken down by autophagy for maintaining the cell viability in *Arabidopsis* (Shibata et al., 2014). Both peroxisome proteins AtPEX6 and AtPEX10 can interact with AtATG8 *via* their own AIMs *in planta* (Xie et al., 2016). An independent yeast two-hybrid screen also identified AtPEX10 as an ATG8-interacting protein (Marshall et al., 2019), suggesting that PEX10 is a promising SAR candidate for pexophagy. Previous study suggested that the Os07g0608800 (termed as OsPEX10) is the homolog of PEX10 in rice (Zolman and Bartel, 2004; Burkhart et al., 2014). Likewise, OsPEX10 has the same AIM (EEYCDI) as AtPEX10 (Xie et al., 2016; Figure 2), so it is speculated that OsPEX10 may bind to ATG8 by this AIM and operate pexophagy-mediated peroxisome quality control in rice. Thus, it sounds that the ATG8-PEX10 driving pexophagy may be highly conserved among plant species.

A LON protease 2 (LON2) is an AAA ATPase thought to prevent pexophagy. The loss of *LON2* caused the decrease of peroxisome number and higher pexophagy (Bartel et al., 2014; Goto-Yamada et al., 2014). Interestingly, if gaining insight to its protein sequences, several putative AIMs appeared and sound reliable, for example, the YLEL predicted as xLIR (Figure 2), implying a possible interaction of LON2 with ATG8. This finding raises a hypothesis that autophagy might also degrade LON2 for releasing its function in driving pexophagy in turn. The protein similarity of LON2 between *Arabidopsis* and rice is more than 80%, suggesting that its function is evolutionarily conserved. Besides, further observation of YVVV AIM in AtLON2 found that the rice one is YIVV (Farmer et al., 2013; Figure 2), suggesting this AIM-based selective autophagy of OsLON2 is also highly conserved.

### ATG8-Interacting Motif Proteins in Chlorophagy

The ATG8-interacting protein 1 (ATI1) and its homolog ATI2 were discovered by a yeast two-hybrid screen in *Arabidopsis*. They contain one predicted transmembrane (TM) domain and two putative AIMs (NEWEVV and ERWQIL) (Avin-Wittenberg et al., 2012; Honig et al., 2012; Figure 2). These proteins are localized on the vesicles, including the ATI1-Endoplasmic Reticulum (ER) bodies and ATI1-Plastid (PS) bodies during carbon starvation or salt stress (Honig et al., 2012; Michaeli et al., 2014). Both vesicles depend on the functional autophagy machinery, but their cargo proteins are likely different. ATI1/2 plays a role in seed germination in response to exogenous abscisic acid (ABA; Michaeli et al., 2014), indicating a link between autophagy, nutrient tolerance, salt tolerance, and ABA. Previous study suggested that the OsATI1/2 (Genebank: NP_001052131.1, NP_001048554.1) is the homolog of *Arabidopsis* ATI1/2 in rice (Honig et al., 2012). Similarly, the conserved AIM (ADWEVV) in OsATI1 is also identified (Figure 2), implying that OsATI1 participates in the selective turnover of specific proteins, such as ABA-associated proteins essential for seed germination. Given that deficiency of ABA biosynthesis would result in preharvest sprouting in rice (Fang and Chu, 2008; Liu et al., 2014), manipulating *OsATI1*-mediated autophagy to orchestrate ABA level or ABA-associated proteins might be a new strategy for overcoming this issue. In addition, ABA is also known to regulate multiple stress response, in particular drought tolerance. Thus, it is also attractive to investigate whether the *OsATI1* is also responsible for the cross talk between ABA and autophagy upon drought. Recently, it was further found that the AIM NEWEVV but not the ERWQIL in the N-terminal of *Arabidopsis* ATI1/2 is functional since mutation of this motif could block the interaction with ATG8 (Sjøgaard et al., 2019; Figure 2). It needs to be emphasized that this AIM is located on the intrinsically disordered regions (IDRs), and thus further supports the notion that AIM present in the IDR is much more reliably functional as previously reported (Kalvari et al., 2014; Mei et al., 2014).

### ATG8-Interacting Motif Protein in Brassinosteroid Signaling

The dominant suppressor of KAR 2 (DSK2) is a SAR containing two regions with high-fidelity AIMs (ESFKEL, EGFNML, and RMYENV) in *Arabidopsis* (Figure 2). Obviously, DSK2 has been verified to interact with ATG8 and target BRI1-EMS Suppressor 1 (BES1) that is essential for brassinosteroid (BR) signaling transduction for autophagic degradation (Nolan et al., 2017). By regulating the BES1 level, DSK2 can alter growth status in response to fixed-carbon starvation or drought stress. Moreover, DSK2 is regulated by the phosphorylation of Brassinosteroid Insensitive 2 (BIN2) kinase (Zhao et al., 2002), and the phosphorylation of DSK2a facilitates the binding to ATG8.

Notably, one homologous DSK2 (termed as OsDSK2a) is reported in rice (Wang et al., 2020). The OsDSK2a is predicted to have two similar AIMs (EGFNML and RMYETV) as AtDSK2 (Figure 2 and Table 2), implying that OsDSK2a may also interact with ATG8 and participate in autophagic degradation of BR signaling-related components, thereby regulating the rice growth under stress. Interestingly, AIMs (EGFNML and RMYETV) of OsDSK2a are also surrounded by BIN2 phosphorylation consensus sites (S/T-X-X-X-S/T) (Zhao et al., 2002; Figure 2), suggesting that activated BIN2 would enhance the interaction between DSK2 and ATG8. Owing to this hypothesis, we propose that BIN2 also indirectly participate in the regulation of autophagy. On another hand, the conserved sequences similarity makes us to hypothesize that OsDSK2a may be also phosphorylated proximal to AIMs by OsBIN2, thereby promoting physical interaction between OsDSK2a and OsATG8. Notably, different from BR-mediated plant phenotypes in *Arabidopsis*, such as dwarfism and the cabbage-like rosette leaf regarding the *bin2/*+ mutants, leaf angle is one of the most obvious traits determined by BR signaling in rice, for instance, *OsBZR1*-RNAi rice displayed erect leaf (Bai et al., 2007). Therefore, it would be quite intriguing to investigate if the rice autophagy mutant would be defect in BR signaling and exhibited a sort of alternation in leaf angle.

**TABLE 2:** Known the ubiquitin-interacting motif proteins in plant.

Species^a^	Protein name (Locus^b^)	Pattern^c^	Annotation	References
*A. thaliana*	RPN10 (NP_195575.1)	NIDPELALALRVSMEEERAR	Degradation of inactive 26S proteasomes	Marshall et al., 2019; Dou et al., 2021
		EDSALLDQAIAMSVGDVNMS		
		DEDQDLALALQMSMSGEESS		
	PUX7 (NP_001077536.1)	EEEEELQRALAASLEDNNMK	Encodes a nuclear UBX-containing protein that can bridge ubiquitin to AtCDC48A	Deruyffelaere et al., 2018; Marshall et al., 2019
	PUX8 (NP_567380.2)	IEEEMIRAAIEASKKEAEGS	Unknown	Ascencio-Ibáñez et al., 2008; de la Fuente van Bentem et al., 2008
		EDDDDIAIAVTMSLKSAEEE		
	PUX9 (NP_680549.3)	AEEEMIRAAIEASKKDFQEG	Unknown	Marshall et al., 2019
		REDEDIARAISMSLEMEEHE		
	PUX13 (NP_567675.1)	EDDDDDDDDDPDYVEEEEEP	Unknown	Gao et al., 2005; de la Fuente van Bentem et al., 2008
*O. sativa*	RPN10 (XP_025876380.1)	NA^d^	Unknown	In this study
	PUX7 (XP_015633410.1)	DEDEELARAVAASLEESKGS	Unknown	In this study
	PUX8 (XP_015612508.1)	NA	Unknown	In this study
	PUX9 (XP_015617568.1)	NA	Unknown	In this study
	PUX13 (XP_015615014.1)	NA	Unknown	In this study

*^a^A. thaliana, Arabidopsis thaliana; O. sativa, Oryza sativa.*

*^b^Gene Locus of the each protein is derived from TAIR (https://www.Arabidopsis.org/index.jsp) and China Rice Data Center (http://www.ricedata.cn/gene/).*

*^c^Pattern is the sequences of the UIM.*

*^d^NA, not applicable.*

### ATG8-Interacting Motif Protein in Iron Metabolism

Another verified SAR with potential AIMs is tryptophan-rich sensory protein (TSPO). TSPOs are membrane proteins that participate in maintaining the concentration of free heme and porphyrins in the plant cells. AtTSPO is transiently induced by ABA and abiotic stresses. The accumulation of TSPO is strictly regulated, with its degradation driven by autophagy using a potential AIM (ALYLYL) (Vanhee et al., 2011; Figure 2). Furthermore, it has also reported that TSPO binds to a plasma membrane-localized aquaporin, plasma membrane intrinsic protein 2;7 (PIP2;7) (Hachez et al., 2015). This interaction facilitates the selective degradation of aquaporin, which inhibits the water uptake in cell. Previous study suggested that OsTSPO (Genebank: XP_015639220.1) is the homolog of TSPO in rice (Jurkiewicz et al., 2020). Interestingly, OsTSPO is predicted to contain AIMs, but they show distinct patterns with those of AtTSPO (Figure 2). Two possibilities regarding this divergence are postulated. First, the interaction of OsTSPO with OsATG8 is loss by the mutation of AtTSPO-like AIM, while other components instead of OsTSPO take in charge of the aquaporin, free heme, and porphyrins metabolism. Alternatively, newly generated AIMs in OsTSPO are still functional for interaction with OsATG8, however, this hypothesis needs to be validated by protein-protein interaction assays, such as yeast two-hybrid and Bimolecular Fluorescent Complimentary (BiFC). Given the less homology between AtTSPO and OsTSPO (less than 40%) (Jurkiewicz et al., 2020), the first view sounds more reasonable that the AIM is discarded from rice due to its dispensable role.

### ATG8-Interacting Motif Protein With Posttranslational Modification

Nitric oxide (NO) is a major cellular signal to modulate plant stress response by *S*-nitrosylated stress-associated proteins at their Cys residue. A newly uncovered mechanism of autophagy in regulating NO signaling has been described recently in *Arabidopsis*, showing that the NO mediator GSNO reductase (GSNOR) is targeted by ATG8 through its AIM (YTVV; residues 152–155; Figure 2), which required *S*-nitrosylation of GSNOR at C-10 residue for exposing this motif to enable its interaction with ATG8 (Zhan et al., 2018). Such nitrosylation-mediated autophagy is operated upon hypoxia response, which is also considered as a remarkable physiological response during submergence. Previously, it has been illustrated that ethylene confers the rice response to waterlogging by *SD1*- or ethylene response factors *SNORKEL1* and *SNORKEL2*-mediated GA signaling and biosynthesis (Hattori et al., 2009; Kuroha et al., 2018), illustrating an important role of ethylene in anaerobic regulation. Recently, it has also been reported that the expression of ethylene responsive regulators, such as *Ethylene Insensitive 2* (*EIN2*) and *EIN3*, was significantly altered in the *Arabidopsis atg* mutants upon submergence, evidently suggesting a cross talk between ethylene and autophagy in response to hypoxia condition (Chen et al., 2015). Considering the GSNOR function in NO signaling, it raises an interesting question that how autophagy, ethylene, and nitrosylation of GSNOR are incorporated to orchestrate the hypoxia-induced NO signaling. On another hand, the rice GSNOR (OsGSNOR) with more than 90% similarity to the *Arabidopsis* one also possesses AIMs (Zhang et al., 2021; Figure 2), among which its YTVV is conserved as AtGSNOR, suggesting that a similar regulation of GSNOR by autophagy is existed among various plants, perhaps due to the importance of anaerobic response for plant survival from submergence. Further exploiting the evolution of nitrosylation underlying GSNOR AIM from algae species to terrestrial plants might provide a new insight into the evolutionary fate of autophagy in NO signaling.

### ATG8-Interacting Motif Protein C53 With Endoplasmic Reticulum-Phagy

Eukaryotes have evolved various quality control mechanisms to promote proteostasis in the ER. Selective removal of certain ER domains *via* autophagy (termed as ER-phagy) has emerged as a major quality control mechanism (Karagöz et al., 2019; Chino and Mizushima, 2020). A recent report identified a cytosolic protein (C53) that is specifically recruited into autophagosomes during ER stress. C53 contains an IDR that bridges two α-helical domains located at the N and C termini. C53 senses proteotoxic stress in the ER lumen by forming a tripartite receptor complex with the ER-associated ufmylation ligase UFL1 and its membrane adaptor DDRGK1 (Gerakis et al., 2019; Stephani et al., 2020). The C53/UFL1/DDRGK1 receptor complex is activated by stalled ribosomes during co-translational proteins translocation and induces the degradation of internal or passenger proteins in the ER. C53 interacts with plant and mammalian ATG8 isoforms *via* a non-canonical AIM, termed shuffled AIM (sAIM) with the consensus sequence “IDWG” (Stephani et al., 2020; Figure 2). Previous study suggested that OsC53 (Genebank: XP_013413714.1) is the homolog of *Arabidopsis* C53 in rice (Stephani et al., 2020). Notably, OsC53 has the similar sAIMs with consensus sequence “IDWD” as the AtC53-IDR (Figure 2), implying that C53-mediated ER-phagy may be a central conserved mechanism operating key quality control of ER homeostasis across various organisms.

### ATG8-Interacting Motif Proteins in Other Biological Processes

As indicated above, the regulation of AIM proteins generally depends on the canonical autophagy. However, there is a contrast case that the ABNORMAL SHOOT3 (ABS3) interacts with ATG8 through two AIMs (WAPL and WVGL) in the late endosome, but the autophagic route is not necessary for such interaction (Jia et al., 2019), thereby defining a novel function of ATG8 independent on autophagy. Notably, further investigation suggested that ABS3 in monocot plants also harbors the two highly conserved AIMs, for instance, in wheat, enabling its interaction with ATG8 (Jia et al., 2019). From this point of view, biological process underlying interaction of AIM proteins with non-autophagic function of ATG8 should be paid much more attentions in the future.

Another recent report showed that the heterotrimeric G-protein complex and regulator of G-protein signaling 1 (AtRGS1) interacts with ATG8a in *Arabidopsis* (Jiao et al., 2019). The study proved that both the N-terminal seven TM (7TM) domain and cytoplasmic RGS domain interact with ATG8, but it is still unknown which domain possess the functional AIMs. This raises a commonly important issue regarding the AIM, why the AIM protein needs several AIMs functioning to interact with ATG8? One possibility is for ensuring the interaction between AIM protein and ATG8 to avoid that one of the AIMs was mutated during evolution and another one could complement. Another possibility is to enhance the interaction affinity and efficiency for rapid response to particular stimuli. Certainly, both assumptions may be tenable, but require further evidence.

Except AIM proteins mentioned above, a part of AIMs of others, but not the proteins themselves, have not been experimentally validated (Table 2). Vierstra group has deduced that the membrane-targeting regulators FYVE2 and FYVE3 likely function to associate autophagosomes to microtubules (Marshall and Vierstra, 2018). The FYVE2 was isolated from an interactome underlying yeast two-hybrid, indicating it interacts with ATG8 in *Arabidopsis*. Previous study suggested that FYVE proteins were predominantly thought to regulate various trafficking pathways (Wywial and Singh, 2010), inspiring the idea that FYVE proteins might bind and deliver ATG8 to relevant location to target the autophagy cargo receptors or cargo proteins.

There are some rest ATG8-interacting proteins identified by protein-protein assay, including chloroplast development regulator Golden 2-Like 1 (GLK1), Kinesin motor family protein KIN7.4, transporter protein GLT1, scaffolding protein of Cajal body COILIN, Nitrate Transporter 1 (NTR1)/Peptide Transporter 2 (PTR2), and Sucrose Transporter 3 (SUC3) in *Arabidopsis* (Arabidopsis Consortium, 2011; Klopffleisch et al., 2011; Chen et al., 2012). Until now, it is still unclear the role of them in autophagic pathway. To this point, it hardly figures out the possible contribution of their homologs to autophagy in rice. However, it still can infer the potential connection of autophagy with them from the insights into their own biological property. For example, the CLAVATA component CLAVATA Complex Interactor 1 (CCI1) is directly induced by WUSCHEL (WUS) and interacts with CLV1 and BAM1 (Gish et al., 2013), which are the key components essential for shoot apical meristem (SAM) homeostasis in *Arabidopsis*. Therefore, it is supposed that autophagy might interfere with SAM development by orchestrating the abundance of CCI1. In conclusion, much more experiments are still required for elaborating the underlying mechanism of selective autophagy of these AIM proteins in various biological processes.

## ATG8-Interacting Motif-Independent ATG8 Interaction: Ubiquitin-Interacting Motif Proteins

Several ATG8-interacting proteins are known to bind ATG8 in an AIM-independent manner (Behrends et al., 2010; Marshall et al., 2015, 2019). One example is *Arabidopsis* RPN10 acting as a cargo receptor for autophagic degradation of the proteasome. RPN10 does not contain any canonical AIM/LIR; instead, three distinct UIMs are present. Among them, UIM1 binds ubiquitin and the UBL domain of DSK2, whereas UIM3 prefers the UBL domain of RAD23, and UIM2 mediated the interaction between RPN10 and ATG8s (Marshall et al., 2015). RPN10 binds to the UDS of ATG8, which is located near the C-terminal glycine on the surface opposite the LDS used by AIM proteins, indicating that AIM and UIM proteins could bind to ATG8 simultaneously (Lin et al., 2013). Similarly, the rice RPN10 homolog (OsRPN10) also has the UIM relevant to the *Arabidopsis* RPN10 (Table 1), suggesting that it also can bind ATG8, consequently conducting proteaphagy. Based on the 19 UDS-specific ATG8 interacting proteins, UIM domain seems to comprise about 20 amino acid length amphipathic α helical structure (Husnjak and Dikic, 2012). By comparison of the verified UIMs, it roughly generates a consensus core sequences for this motif containing invariant alanine and serine residues, Ψ-ζ-X-A-Ψ-X-X-S, in which the Ψ, ζ, and X represents small hydrophobic residues, hydrophilic residues, and any amino acid, respectively (Hofmann and Falquet, 2001; Marshall et al., 2019).

Another case of UIM protein is the plant ubiquitin regulatory X domain (PUX) proteins, which bind ATPase CDC48 (p97 in humans) to regulate the latter one’s assembly and activity, leading to diverse cellular activities (Rancour et al., 2004; Gallois et al., 2013). Four PUXs (PUX7, PUX8, PUX9, and PUX13) out of the PUX family interact with ATG8 *via* UIMs, thereby acting as SARs turning over the non-functional CDC48 complexes (Marshall et al., 2019). Regarding the acquisition of the UIM protein sequence in rice, a homologous comparison was carried out by using the known *Arabidopsis* UIM proteins as query correspondingly. The resulted homologs in rice were isolated by SMART of the conserved domains (Letunic and Bork, 2018; Letunic et al., 2021), and then selected for the comparisons of UIMs between the *Arabidopsis* and rice. As a result, the UIM of OsPUX7 can be detected but OsPUX8 cannot, whereas the PUX9 and PUX13 are notably absent in rice (Table 1). It is possible that OsPUX7 binds to ATG8 through a UIM to mediate the autophagic degradation of CDC48 complexes, which is adequate to replace the roles of other PUX proteins in selective autophagy.

Previously, the clathrin Adaptor Protein-1 Mu-adaptin 2 (AP1M2) and ER-localized co-chaperone B-cell lymphoma 2 (Bcl-2)-Associated Athanogene 7 (BAG7) have not shown to be associated with ATG8 and/or autophagy (Park et al., 2013; Nawkar et al., 2018). However, they may still function as specific cargo receptors or adaptors for autophagic vesicle dynamics, because the putative UIMs are found in both OsAP1M2 and OsBAG7 but not the *Arabidopsis* ones (Marshall et al., 2019), implying that UIMs of AP1M2 and BAG7 are evolutionarily differentiated between dicotyledonous and monocotyledonous plants. Therefore, we assume that this kind of genetic variation causes a diversified evolutionary fate of such UIM protein-dependent autophagy within plant species.

## Future Perspectives

**FIGURE 3:**
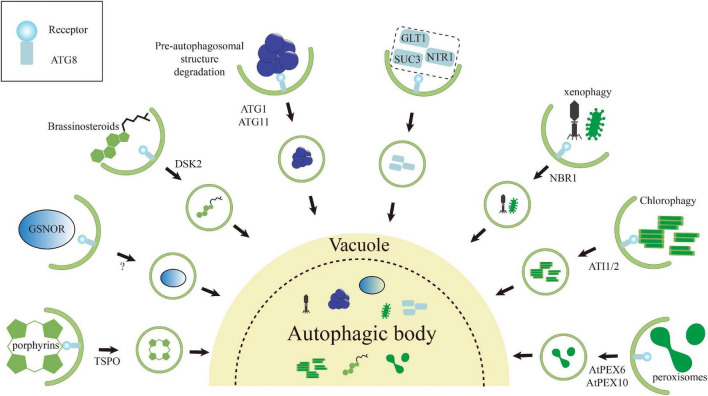
Schematic representation of various selective autophagy pathways known in plants. The autophagic degradation routes for organelles, protein complexes, protein aggregates, and pathogens are shown, and unique features of each are highlighted. Numerous examples of intracellular pathogens being degraded by autophagy (called xenophagy), such as NBR1-mediated elimination of the cauliflower mosaic virus (CaMV) P4 protein. Interestingly, the turnip mosaic virus (TuMV) could induce unfolded protein response (UPR)-dependent NBR1-ATG8 autophagy to enhance virus propagation to protect itself. Degradation of chloroplasts *via* chlorophagy may occur through the formation of ATI1/2-decorated bodies containing plastid membrane proteins from either the outer envelope or thylakoids during the carbon starvation or salt stress. Both peroxisome proteins PEX6 and PEX10 can interact with ATG8 *via* their own AIMs, suggesting that these proteins may trigger pexophagy to maintain the cell validity. Both ATG1 and ATG11 contain an AIM that likely connect ATG1 kinase complex to the pre-autophagosomal structure. The interaction may not only help the pre-autophagosomal structure assembly, but also promote its autophagic turnover. Degradation of tryptophan-rich sensory protein (TSPO) by selective autophagy has been supposed as an iron metabolic mechanism to effectively clear porphyrins in cell and to control the water uptake by selective removal of plasma membrane aquaporins during osmotic stress. The brassinosteroid (BR) signaling transcription factor BES1 is degraded by selective autophagy upon ubiquitylation and delivery to the phagophore by DSK2, with binding to the ATG8 regulated by BIN2-mediated phosphorylation around its AIMs. NO-mediated *S*-nitrosylation of GSNOR because local conformational changes that render its AIM accessible for ATG8, thereby inducing selective autophagy of GSNOR to regulate hypoxia responses. Some rest ATG8-interacting proteins, including GLT1, NTR1/PTR2, and SUC3 in *Arabidopsis*.

The plants conduct various strategies to conquer challenges from changing environmental conditions. Autophagy is one of the most robust strategies, which is highly conserved in eukaryotes and involved in plant development and stress responses. However, there are still many opening questions regarding the biological significance of autophagic degradation of specific targets (Figure 3). ATG8 is one of the best-studied proteins of the core autophagy machinery. In selective autophagy, ATG8 proteins play dual roles in the formation of autophagosome and recruitment of SARs, respectively. The latter role of ATG8 is generally dependent on the AIM/UIM present in the SARs. Absolutely, identification of the SARs enables to dissect the specific function of autophagy in relevant biological processes. The iLIR and hfAIM predicting AIM are available for this goal. Since there are less UIM proteins verified experimentally, it is hard to conclude a regular pattern of its canonical sequences, limiting the high-throughput screening of UIM proteins by bioinformatics. Although the two available bioinformatic approaches are powerful for identifying AIM, false positives still cannot be avoided. Therefore, more specific and stringent criteria are still pursued. The location of AIM in disordered region can be considered as one reference index. However, it has to keep in mind that a part of AIM proteins would expose its AIM after posttranslational modification, such as the GSNOR mentioned above, or the interaction affinity of them with ATG8 would be strengthened by conformational changes, such as negative charge form surrounding AIM. Therefore, bench work is still needed for confirmation. Recently, the AlphaFold has been developed for predicting the protein structure with enormous fidelity (Jumper et al., 2021; Tunyasuvunakool et al., 2021), which would potentially improve the identification of AIM and UIM based on the structural conservation of verified ones. Comparison of the conservation of AIM among different plant species can facilitate our knowledge of undefined regulatory mechanism of autophagy in certain organisms. Notably, if there is no homolog of AIM/UIM proteins among different plant species, it is also quite valuable for exploring the evolutionary differentiation of autophagy regarding these AIM/UIM proteins.

It has to remind that plant ATG8 family proteins generally consist of multiple isoforms, such as there are nine isoforms in *Arabidopsis* and seven in rice (Kwon and Park, 2008; Xia et al., 2011). Though it has been proposed that these isoforms would function redundantly, it is still deserved to dissect their divergent functions in different biological processes. The specific spatial and temporal expression pattern of them might determine which cargo receptors or cargo proteins would be recruited by corresponding ATG8 individual. Considering autophagy degrades proteins, broad proteomic profiling of autophagy mutants and *ATG*-overexpression lines might provide candidates associated with selective autophagy. Integrative analysis of AIM/UIM present in such proteins could further support the fidelity of them. Although so many AIM/UIM proteins have been extensively characterized, it is very interesting to ask if there are still numerous proteins independent on these motifs for an unknown route underlying selective autophagy. The establishment of novel methods to monitor and investigate autophagy *in planta* would be helpful for screening and deciphering such components. Nonetheless, more than one AIM/UIM might be located in some AIM/UIM proteins, such as ABS3 harboring two functional AIMs mentioned above, which might complement the effect of each other. In this scenario, multiple mutations of them are all necessary for elucidating the underlying mechanism.

In summary, autophagy plays a pivotal role in maintaining cellular homeostasis. This degradation regulation ensures the clearance of macromolecules, including protein complex, large aggregate, and even the entire pathogen and organelle. Furthermore, it also functions as positive rather than passive effect by recycling nutrients for new issues and organs development under stressful condition, such as seed, eventually ensuring plants survival, and transmitting progeny. Crop yield and productivity have been adversely threatened by recent extreme climate change and environmental stresses. To feed the growing population worldwide, genetic improvement and breeding of new crop varieties with higher yield upon suboptimal conditions are indeed desirable, and thus manipulation of autophagy may be a useful strategy for tackling this task.

## Author Contributions

QX and WL conceived the idea. QX, WL, and ZL led the writing of the manuscript. ZL and ZM helped in revising the manuscript and prepared the figures. QX, WL, SG, and YL reviewed, edited, and improved the manuscript. All authors contributed to the article and approved the submitted version.

## Conflict of Interest

The authors declare that the research was conducted in the absence of any commercial or financial relationships that could be construed as a potential conflict of interest.

## Publisher’s Note

All claims expressed in this article are solely those of the authors and do not necessarily represent those of their affiliated organizations, or those of the publisher, the editors and the reviewers. Any product that may be evaluated in this article, or claim that may be made by its manufacturer, is not guaranteed or endorsed by the publisher.
